# Valorization of lithium extraction by-products: efficient arsenic adsorption using synthetic aluminosilicates

**DOI:** 10.1039/d5ra05708f

**Published:** 2025-10-20

**Authors:** Juan C. Villafranca, Daniel Rosales, Marcelo Esquivel, Mario H. Rodriguez, Tamara A. Altieri, Nicolás Arancibia, Estefanía M. Martinis

**Affiliations:** a Facultad de Ingeniería, Universidad Nacional de Cuyo – Centro Universitario Ciudad de Mendoza M5500 Mendoza Argentina emartinis@mendoza-conicet.gob.ar estefania.martinis@gmail.com +54-261-4672305; b Consejo Nacional de Investigaciones Científicas y Técnicas (CONICET) Argentina; c Facultad de Ciencias Exactas y Naturales, Universidad Nacional de Cuyo, Consejo Nacional de Investigaciones Científicas y Técnicas (CONICET) Mendoza Argentina; d Centro Atómico Bariloche, (CNEA – CONICET) Av. Bustillo km 9.5 C.P.8400 Bariloche Argentina; e Universidad Nacional del Comahue (UNCo) Bariloche Quintral 1250 CP 8400 Bariloche Argentina; f Gerencia Química, GASNyA, CAC – Comisión Nacional de Energía Atómica Av. Gral. Paz 1499, San Martín Buenos Aires Argentina; g Center for the Development of Nanoscience and Nanotechnology, CEDENNA Santiago Chile; h Facultad de Química y Biología, Universidad de Santiago de Chile Santiago Chile

## Abstract

The contamination of water resources by heavy metals, particularly arsenic, represents a critical environmental and public health challenge globally. Arsenic, originating from both geogenic processes and anthropogenic activities such as mining and industrial discharges, accumulates in aquatic systems, posing severe risks due to its toxicity. This study investigates the adsorption efficacy of synthetic aluminosilicates, specifically albite and nepheline, synthesized as by-products from an innovative lithium extraction process, for the remediation of arsenic-contaminated water. A comprehensive suite of characterization techniques, including XRD, SEM-EDS, FTIR, zeta potential, BET, adsorption kinetics, and isotherms, was employed to thoroughly evaluate the adsorbent materials. The research delineates the influence of solution pH and adsorbent dosage on the arsenic adsorption capacity of these materials. Comprehensive analyses of adsorption mechanisms and adsorbate–adsorbent interactions were conducted to elucidate the underlying processes. The results of this study demonstrate the potential of these synthetic aluminosilicates as efficient, low-cost adsorbents for arsenic removal, offering a viable and sustainable approach to address arsenic pollution in water resources. The innovative aspect of utilizing by-products from lithium extraction not only provides a novel solution for arsenic remediation but also adds value to industrial waste, promoting a circular economy. The findings have significant implications for the development of scalable and eco-friendly water treatment technologies, addressing both environmental sustainability and public health concerns.

## Abstract 2

Synthetic aluminosilicates obtained from lithium extraction by-products exhibit strong arsenic adsorption performance, highlighting their potential for sustainable water purification and circular economy applications.

## Graphical abstract

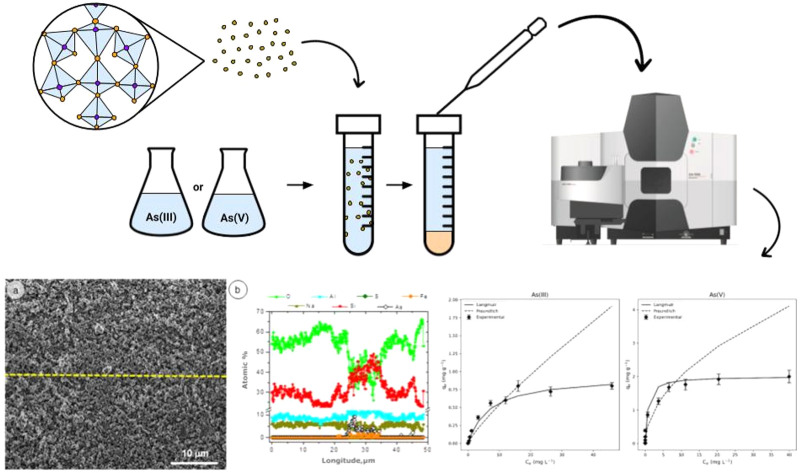

## 1. Introduction

The contamination of water bodies by heavy metals is currently a growing environmental problem.^1^ Heavy metals in water resources originate from both natural and anthropogenic sources. Among the natural ones, we can name atmospheric depositions and water/soil and water/rock interactions. On the other hand, anthropogenic sources can include industrial, mining, agricultural, and urban activities.^2^ The treatment and removal of these types of contaminants is an immediate need since they represent a risk to ecosystems and human life due to their bioaccumulation and toxicity.^3^

Arsenic is a metalloid that causes toxic effects in humans and ecosystems.^4^ The presence of arsenic (As) in the environment comes from both geogenic sources and human economic activities.^5^ Among natural factors, arsenic levels in water bodies largely result from the natural dissolution of minerals from eroded rocks and soils, primarily iron oxides or sulfide minerals. Specifically, the main natural source of arsenic in contaminated aquifers is arsenic-rich minerals such as arsenopyrite, arsenian pyrite, and enargite.^6^ Among the anthropogenic sources we can find the combustion of fossil fuels, particularly coal, which introduces large amounts of arsenic into the environment, much of which reaches natural waters. Other sources include arsenic-based agrochemicals (insecticides, pesticides, and fertilizers), as well as waste from mining, smelting, and tanning industries.^7,8^ The toxicity of arsenic varies significantly depending on its chemical form, with inorganic arsenic being more toxic than organic arsenic.^9^ The specific form of arsenic present in groundwater is influenced by various factors, including climatic conditions, geological and tectonic settings, hydrogeochemical parameters such as pH, redox potential, ionic strength and concentrations, as well as the presence of organic matter and microbial activity.^10^ In aqueous environments, arsenic is predominantly found in its inorganic forms, arsenite As(iii) and arsenate As(v).^11^

Arsenic contamination in groundwater can lead to a range of health issues, including circulatory and digestive problems, diabetes, skin conditions, among others. Research has also linked arsenic-tainted drinking water to an increased risk of cancer.^12,13^

Physical, chemical and biological methods have been applied to the removal of arsenic in aqueous media.^14^ These methods have disadvantages such as waste generation and high costs.^15^ On the other hand, adsorption techniques constitute an adequate alternative in the removal of heavy metals due to their high efficiency, low costs and ease of operation.^16,17^

Adsorption is a mechanism by which atoms or molecules (adsorbates) are superficially attracted to liquids or solids (adsorbents).^17^ This technique has been widely applied in the treatment of water contaminated with organic and inorganic compounds.^18^ Due to the great variety of contaminants, different materials have been used as sorbents, among which we can name clay minerals, oxides, agricultural and industrial by-products, among others.^19,20^

Although there are a wide variety of adsorbents, not all materials have the ability to retain metal anions.^21^ Aluminosilicates are low-cost adsorbents compared to other materials and have been used in the removal of contaminants due to their structure and composition.^22^ A class of aluminosilicates, tectosilicates, are defined by dense tetrahedrons of Al or Si that form a crystalline structure in the presence of sodium, potassium, and calcium. Quartz, zeolites, scapolites, feldspathoids, and feldspar are groups that constitute the larger category of tectosilicates.^23^

Feldspars constitute the most abundant mineralogical group in the Earth's crust, accounting for nearly 58% of its composition. Nevertheless, their applications have been largely confined to industrial uses, particularly in the ceramic and glass sectors, where they serve as fluxing agents in the production of stoneware, porcelains, bricks, and glass-ceramics. In contrast, their potential in environmental remediation, such as in water treatment for the removal of toxic contaminants, remains largely unexplored, highlighting the novelty of employing feldspar-based materials in adsorption processes.^23^ The structural and chemical properties of albite (NaAlSi_3_O_8_) and nepheline (NaAlSiO_4_), both types of aluminosilicates, make them versatile materials for adsorption applications. This versatility is demonstrated in the work of Rosales *et al.*,^24^ who showed that albite and nepheline can be produced as by-products in a novel lithium extraction method. This process involves the dry fluorination of α-spodumene ore with sodium fluoride, yielding albite, nepheline, and lithium fluoride while reducing energy consumption compared to traditional extraction methods. These synthetic aluminosilicates offer a significant opportunity for repurposing in environmental applications.^24–26^

The global demand for lithium has surged dramatically in recent years, driving a rapid expansion of mining and extraction activities, with production expected to continue increasing significantly in the coming decades. However, these operations are associated with notable environmental impacts, including water pollution from mining waste, loss of biodiversity, and ecosystem destabilization due to large-scale land use and chemical contamination.^27^ Although specific evidence on arsenic release from lithium mining is lacking, this industry is known to contribute to the emission and transport of various toxic species and to generate substantial amounts of waste.^28^ In this context, the approach of our work is particularly relevant, as it explores the use of a mining by-product for arsenic removal, with potential applications to other contaminants as well.

Building on these findings, the present study focuses on the potential use of albite and nepheline as adsorbents for arsenic removal. The study includes a comprehensive suite of characterization techniques, including XRD, SEM-EDS, FTIR, zeta potential, BET, adsorption kinetics, and isotherms, to thoroughly evaluate the adsorbent materials. By investigating their adsorption capacity under varying pH levels and adsorbent concentrations, this research aims to enhance our understanding of their environmental applicability and the mechanisms that influence their interaction with arsenic in aqueous solutions.

## 2. Materials and methods

### 2.1. Solutions preparation

Arsenic trioxide (As_2_O_3_, 99.3%, Biopack), sodium arsenate heptahydrate (Na_3_AsO_4_·7H_2_O, 98.0–102.0%, Biopack), sodium hydroxide (NaOH) and nitric acid (HNO_3_, Anedra) were used to prepare solutions. Stock solutions of As(iii) and As(v) of 1000 mg L^−1^ were prepared by dissolving As_2_O_3_ and Na_3_AsO_4_·7H_2_O respectively in distilled water. Working solutions of different concentrations were prepared by dilution. The chemical stability of aqueous As(iii) and As(v) species has been extensively documented, with studies demonstrating that both forms remain stable over experimental periods when solutions are freshly prepared and maintained under controlled laboratory conditions.^29,30^

### 2.2. Adsorbent

The materials used for the adsorption tests correspond to mixtures of albite (NaAlSi_3_O_8_) and nepheline (NaAlSiO_4_) generated as by-products in obtaining lithium from the mineral α-spodumene (α-LiAlSi_2_O_6_).^24^ The adsorbents were synthesized in-house by applying a dry fluorination method to α-spodumene.^24–26^eqn (1) (ref. 24 and 25) describes the predicted reaction during the roasting of the mixture of spodumene and NaF.

1 2α-LiAlSi_2_O_6_ + 2NaF → 2LiF + NaAlSi_3_O_8_ + NaAlSiO_4_

The obtained mixture underwent a washing process with distilled water and dissolution in H_2_SO_4_.^26^ Consequently, Li^+^ remains in solution for subsequent recovery as a commercial Li salt, while the albite and nepheline compounds are separated for use as adsorbent material (non activated sample – (NA)).LiF_(s)_ + NaAlSi_3_O_8(s)_ + NaAlSiO_4(s)_ + H_2_SO_4(aq)_ → Li_(aq)_^+^ + H_2_SO_4(aq)_ + NaAlSi_3_O_8(s)_ + NaAlSiO_4(s)_

The non activated sample (NA) was placed in a planetary mill (Retsch 100) with a chamber and stainless-steel balls in an air atmosphere. The sample was subjected to a grinding process at different times (15, 120, 240, 420 and 600 minutes) at a constant rotation speed of 500 rpm obtaining samples (A1, A2, A3, A4, A5).

### 2.3. Material characterization

X-ray diffraction analysis of the adsorbents was conducted using a PANalytical Empyrean system with Cu-Kα radiation at 40 kV and 30 mA. For morphological and elemental characterization, a FEI INSPECT s50 scanning electron microscope coupled with an EDAX – Octane Pro energy dispersive X-ray spectrometer was employed. Fourier transform infrared spectroscopy (FTIR) spectra of KBr pellets were obtained using a FT-IR spectrometer PerkinElmer Spectrum 100.1 mg of sample was mixed with 100 mg of KBr. The FTIR spectra ranged from 450–4000 cm^−1^. The isoelectric point (IEP) or point oz zero charge was obtained from the pZeta *vs.* pH plot using a Zetasizer 2000 Marlvern. In both dispersions, the ionic strength was kept constant (KCl 10 mM), the desired pH value was set using HCl or KOH as appropriate, the system was equilibrated for 10 minutes and the zeta potential was measured. The textural properties of the samples were determined by nitrogen adsorption at a temperature of 77 K using the Micromeritic Gemini V2.0 model 2380 equipment. Prior to this, all samples were degassed at a constant temperature of 130 °C for approximately 12 hours using a Micromeritics Flow Prep 060 instrument. The BET model was employed for specific surface area determination. Micropore area and micropore volume were determined using *t*-curves (Halsey equation). Pore size distribution and pore size were determined according to the model proposed by Barrett, Joyner, and Halenda (BJH).

The As determination was performed with a SHIMADZU AAnalyst 7000 Atomic Absorption Spectrophotometer, Series A using the instrumental technique of Hydride Generation coupled to Atomic Absorption Spectrometry.

### 2.4. Sorption experiments and effect of different parameters on arsenic removal

Batch adsorption experiments were conducted to assess the material's ability to remove arsenic. Initially, 0.1 g of the material was placed in 15 mL Falcon test tubes with screw caps (PP). Subsequently, 10 mL of 1 mg L^−1^ solutions of As(iii) and As(v) were added to each tube, which were then immersed in an ultrasonic bath for 10 minutes. The tubes were shaken at 1000 rpm at room temperature for 60 minutes. After the adsorption process, the samples were centrifuged at 5000 rpm for 30 minutes to separate the phases. The supernatants were then carefully pipetted out using Pasteur pipettes for arsenic concentration analysis. Arsenic concentration was determined in the aqueous phase remaining after adsorption experiments, using HG-AAS. The removal efficiency was calculated using eqn (2):2
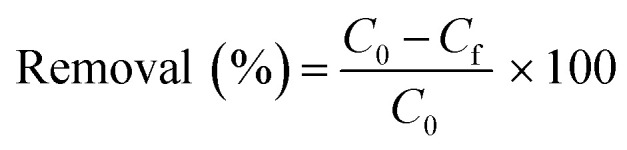


The following experiments were designed to evaluate the performance of adsorbents under various conditions, focusing on the effects of mechanical activation, adsorbent mass, pH, kinetics, and equilibrium isotherms.

#### 2.4.1. Effect of mechanical activation

The impact of mechanical activation on arsenic removal was evaluated using 0.1 g of each sample (A1, A2, A3, A4, A5), following the procedure previously described.

#### 2.4.2. Effect of adsorbent mass and pH

The mass of adsorbent and solution pH were examined as critical parameters affecting arsenic removal. Experiments were conducted with adsorbent masses of 0.01, 0.05, 0.1, and 0.2 g in 10 mL of solution. To assess the pH effect, working solutions with pH values ranging from 3 to 11 were prepared, adjusting with HNO_3_ and NaOH as needed.

#### 2.4.3. Adsorption kinetics

Kinetic studies were performed to determine the equilibrium time for arsenic adsorption. For this, 0.1 g of activated adsorbent was mixed with 10 mL of a 1 mg L^−1^ solution containing As(iii) and As(v) at an initial pH of 8. The dispersions were agitated at 1000 rpm and 25 °C for various durations (1, 5, 10, 30, 60, 90, and 120 min). After centrifugation for 30 min, the supernatants were analyzed to quantify the remaining arsenic concentration.

#### 2.4.4. Adsorption isotherms

Equilibrium studies were conducted to quantify the adsorption capacity of the adsorbent. Batch experiments were performed by mixing 0.1 g of adsorbent with 10 mL of solutions of known As(iii) and As(v) concentrations at pH 9. The dispersions were agitated for 1 hour at 25 °C to reach equilibrium, then centrifuged to collect supernatants. The arsenic concentration in the supernatants was measured using atomic absorption spectroscopy, and the adsorbed arsenic was calculated from the difference between the initial and final concentrations.

Sorption isotherms were used to model the interaction between the adsorbent and arsenic species. Parameters derived from these isotherms provide insight into the surface properties of the adsorbent and its affinity for arsenic in solution.^31^

## 3. Result and discussion

### 3.1. Material characterization

To compare changes due to mechanical activation of the sample, the non-activated sample (NA) and the sample with the longest activation time (A5) were analyzed.

**Fig. 1:**
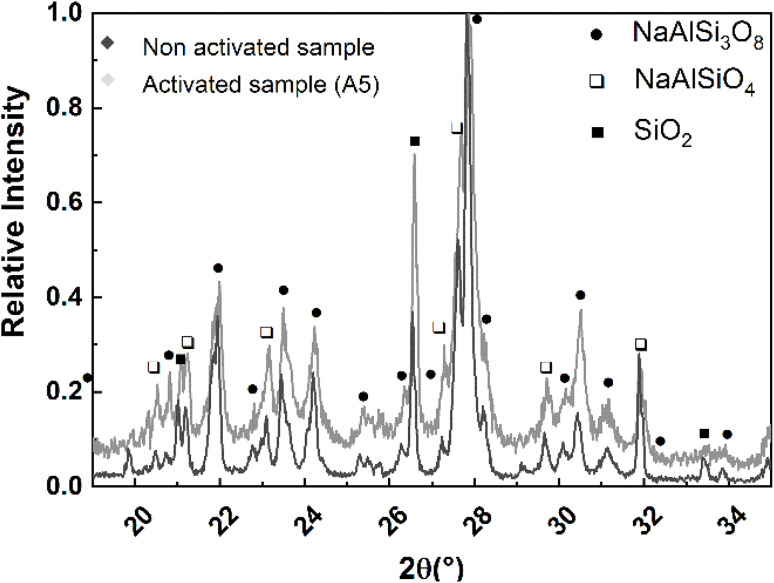
XRD pattern of non activated and A5 sample.

XRD patterns of albite, nepheline, and quartz are shown in Fig. 1. The diffractograms correspond to the non-activated sample (gray) and the sample A5 with the longest activation time (light gray). The width of a reflection peak depends on the perfection of the crystal and its size, as the mean size of the crystals increases, the angular extent of the peak (2*θ*) decreases. In the pattern of the activated sample, a broadening of the peaks is observed, which indicates smaller crystals in relation to the non-activated sample. The presence of characteristic peaks of impurities or broad peaks corresponding to amorphous phases is not observed.

**Fig. 2:**
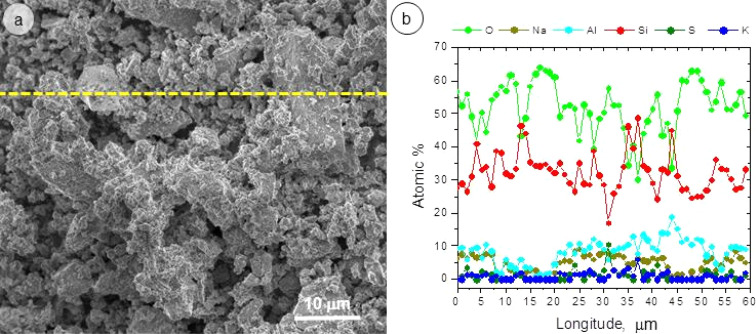
(a) SEM micrograph and (b) EDS line scan of non activated sample.

**Fig. 3:**
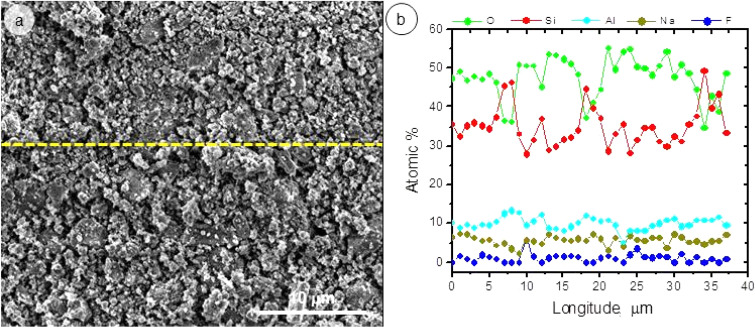
(a) SEM micrograph and (b) EDS line scan of A5 sample.

Fig. 2 and 3 show the SEM micrographs and the line scan corresponding to Energy Dispersive X-ray Spectroscopy (EDS) over the non activated and A5 sample.

The results presented in Fig. 2 and 3 show that an increase in milling time produces a marked decrease in the particle size of the aluminosilicates. The particle size decreases from ∼10 μm (non activated sample) to ∼1 μm (activated sample). On the other hand, EDS analysis reveals that the sample is composed of Na, Al, Si, and O in varying amounts. This is consistent with the XRD analysis, which detects the phases NaAlSiO_4_ and NaAlSi_3_O_8_.

**Fig. 4:**
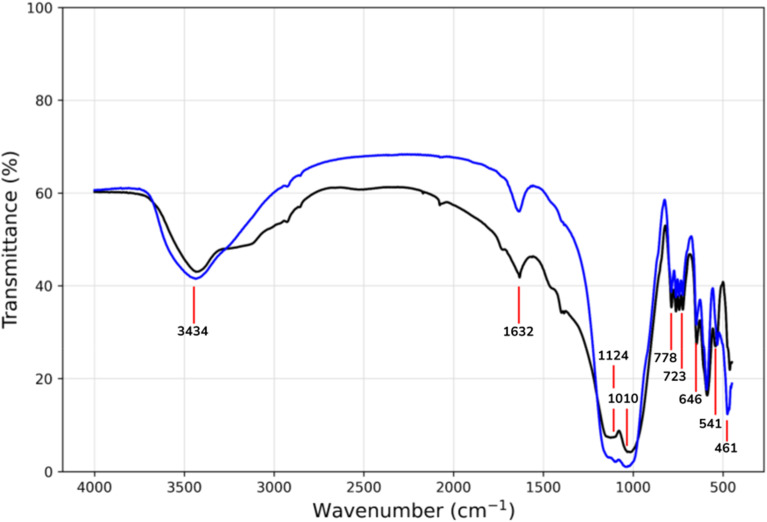
FTIR spectra of sample NA (black) and A5 (blue).

The FT-IR spectroscopy on the mixture sample was conducted and their corresponding spectra are shown in Fig. 4. The sample revealed a pattern of IR spectra similar to that described in the literature.^32–34^ The spectra showed many peaks in the region below 1300 cm^−1^. The principal infrared characteristic peaks of albite and nepheline are observed in the 400–1300 cm^−1^ range.^35^ Peaks around 541 and 461 cm^−1^ represented the bending vibrations of Al–O–Si and Si–O–Si, respectively. The peak at 646 cm^−1^ corresponds to the combined out-of-plane vibrations of Al–O and Si–O. The bands at 1010 cm^−1^ and 723 cm^−1^ are associated with the asymmetric and symmetric stretching vibrations of the aluminosilicate framework (Si–O–Al), respectively. The band at 1124 cm^−1^ is linked to the stretching vibration of Al–O. The band at 778 cm^−1^ is attributed to the bending vibrations of Si–O–Si. The peaks at 1632 cm^−1^ and 3434 cm^−1^ are related to the bending and stretching vibrations of water molecules.

#### 3.1.1. Point of zero charge

**Fig. 5:**
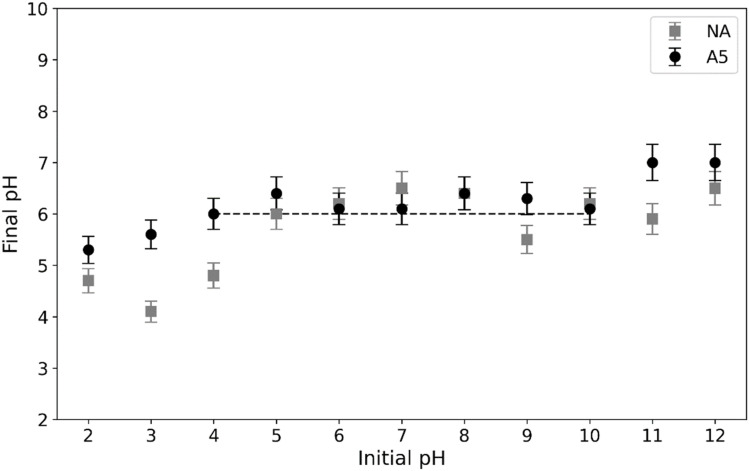
pHpzc determination. Experimental conditions: sorbent dose: 0.1 g, contact time: 24 h.

The point of zero charge (pHpzc) represents the pH at which the net surface charge of an adsorbent is zero, a critical parameter in adsorption studies as it determines the likelihood of physisorption.^36^ The pHpzc can be determined by plotting equilibrium pH values against initial pH, with the plateau in the graph indicating the pHpzc. Samples NA and A5 exhibit similar pHpzc values (5–7), consistent with previous findings for albite (pHpzc ∼ 6.8), which attribute this value to the protonation and deprotonation of oxygen atoms in Al tetrahedrons (Fig. 5).^37^

Reported differences in literature may arise from compositional variations and impurities. Several factors influence the pHpzc, including crystalline structure, Si : Al ratio, impurity content, and ion adsorption characteristics.^36^

**Fig. 6:**
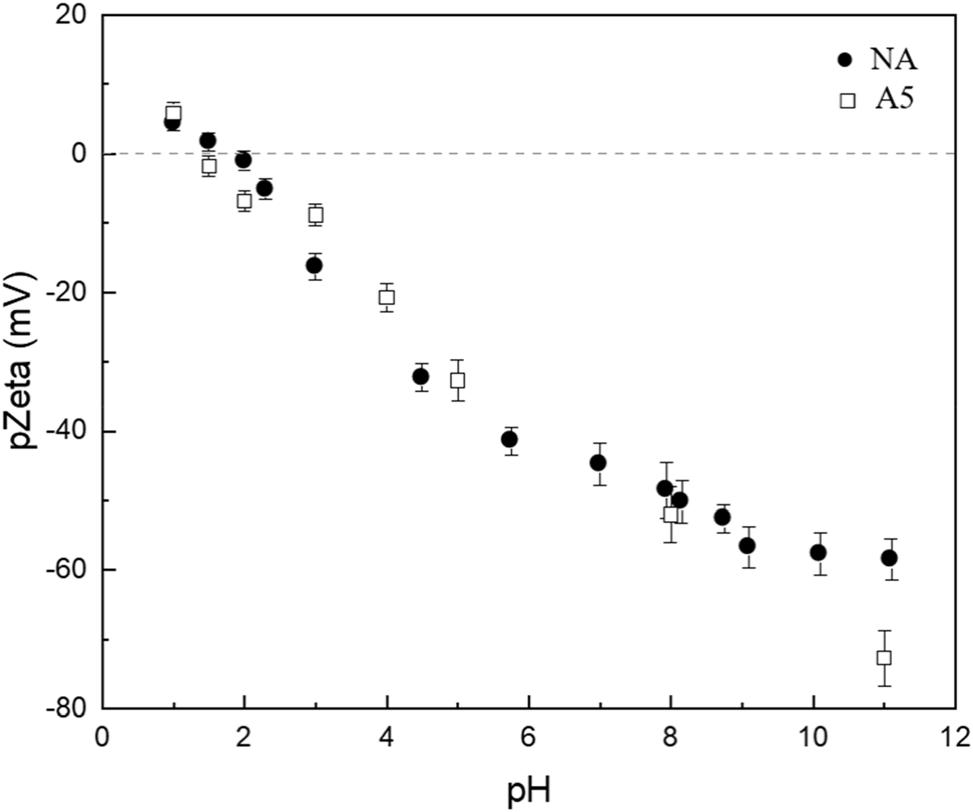
Zeta potentials of NA and A5 as a function of pH.

In Fig. 6, the graph of electrophoretic mobility as a function of pH is shown, determining that the isoelectric point (IEP) value for both substrates is approximately 1.5. The differences observed between the point of zero charge (pHpzc) and the IEP are due to these parameters reflecting different surface conditions. The pHpzc corresponds to the pH at which the total net charge (internal and external) of a material's surface is equal to zero, indicating an equilibrium between positive charges (protonated) and negative charges (deprotonated). In contrast, the IEP is associated with the pH where the electrophoretic mobility of the substrate, predominantly determined by its external surface, is zero. Previous studies have indicated that the leaching of sodium and aluminium from albite modifies the composition of its external surface, which explains the differences between the two parameters. Evidence reports isoelectric points as low as 1 for albite, where the formation of an altered layer rich in silicon shifts the IEP toward significantly more acidic conditions.^37^ In addition, previous work has demonstrated that compositionally similar aluminosilicates undergo selective leaching of Na^+^ and framework Al under acidic conditions, leading to the development of a Si-rich external surface.^26,38^

**Fig. 7:**
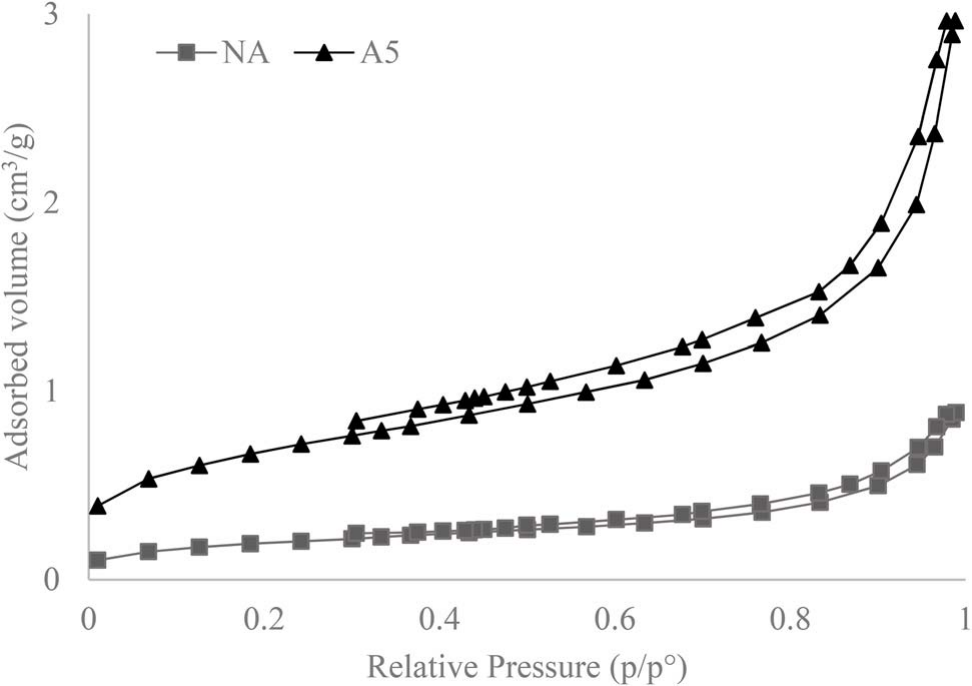
Nitrogen adsorption/desorption isotherms for NA and A5 obtained at −196 °C (77 K).

**Fig. 8:**
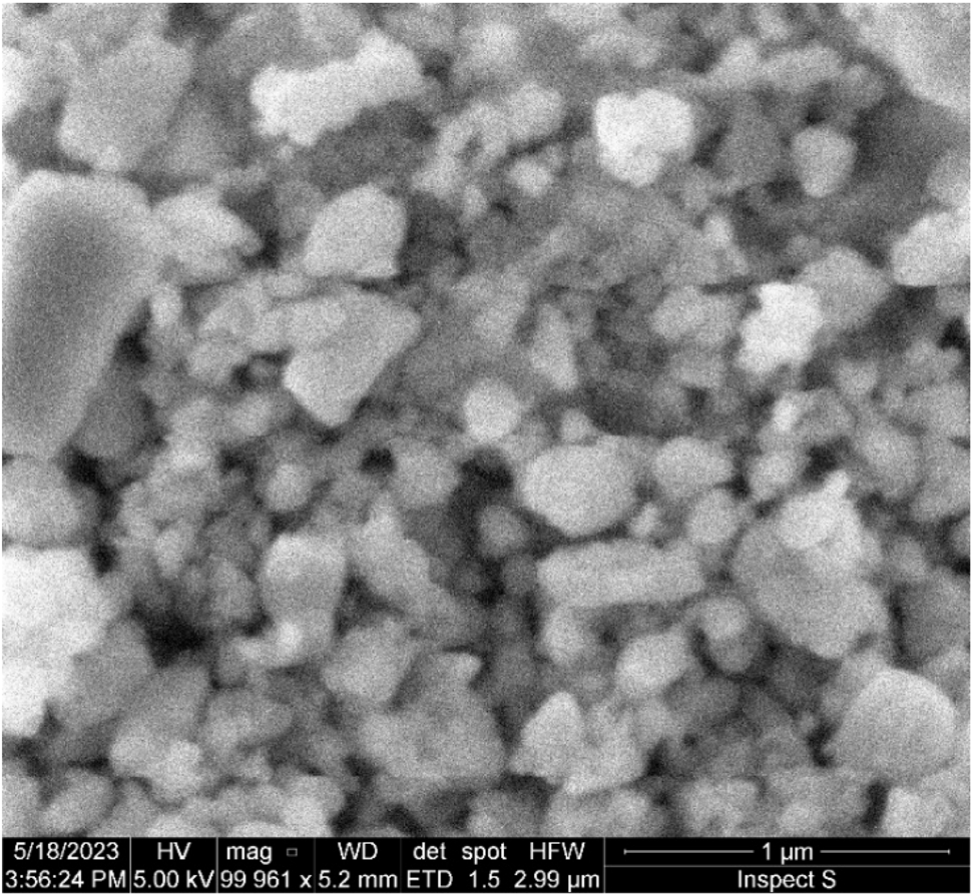
SEM micrograph of A5.

Textural analyses were conducted on two samples, the NA sample and the sample with longer activation time, A5. The obtained isotherms for the NA and A5 samples correspond to Type II isotherms according to the IUPAC classification, which is characteristic of solids with heterogeneous texture, exhibiting H3 hysteresis (Fig. 7). A Type II isotherm is characteristic of macroporous materials or solids with a heterogeneous texture, meaning they have a wide pore size distribution, ranging from relatively small pores to larger pores. This suggests that the material possesses a combination of micropores (nanosized pores) and mesopores (intermediate-sized pores, typically in the range of 2 to 50 nanometers). In an H3 hysteresis, adsorption occurs more rapidly than desorption, and the desorption curve is located below the adsorption curve at intermediate relative pressures. According to IUPAC, the H3 loop is frequently associated with slit-shaped pores generated by non-rigid aggregates of lamellar particles; however, it may also originate from interparticle porosity in materials composed of non-lamellar grains. In our SEM micrographs (Fig. 8), the solid appears as hierarchical aggregates of submicrometric particles (∼50–200 nm) without evident lamellar morphology. The porous texture arises from the loose packing of these grains and from their linear contact points, which generate slit-like voids and wedge-shaped pores at the interparticle necks. This morphology is consistent with the H3 hysteresis (absence of closed cylindrical mesopores and presence of open inter-aggregate porosity) and with the Type II character of the isotherm, where the external surface area predominates and adsorption increases continuously at high relative pressures (*P*/*P*_0_) without reaching a well-defined plateau. In summary, although stacked plate-like particles are not observed, the network of interparticle slits formed by the albite/nepheline aggregates accounts for the H3 hysteresis observed.

Table 1 displays the obtained results for the BET specific surface area, micropore surface area, pore volume, and pore size. The BJH pore size distribution reveals the predominant existence of pores in the mesoporous region with a pore size between 8-10 nm. The values obtained for external and microporous areas lead to the conclusion that the material's surface is primarily external, as the micropore volume obtained using the t-method is very low, indicating a lack of microporosity.^39^

**Table 1:** Specific surface are, pore volume and pore size of Na and A5

Sample	BET surface area (m^2^ g^−1^)	*t*-Plot micropore area (cm^3^ g^−1^)	Pore volume (cm^3^ g^−1^)	Pore size (nm)
NA	2.35 ± 0.12	0.14 ± 0.01	0.004 ± 0.0002	10.37 ± 0.52
A5	8.29 ± 0.41	0.18 ± 0.01	0.015 ± 0.0008	8.49 ± 0.42

Regarding the effects of activation, the results confirm that mechanical activation increased the specific surface area and pore volume by almost 4 times while decreasing the pore size by 20%. These findings indicate that mechanical activation has had a significant impact on the textural properties of the A5 sample compared to the NA sample.

### 3.2. Sorption experiments

#### 3.2.1. Activation effect on arsenic adsorption

**Fig. 9:**
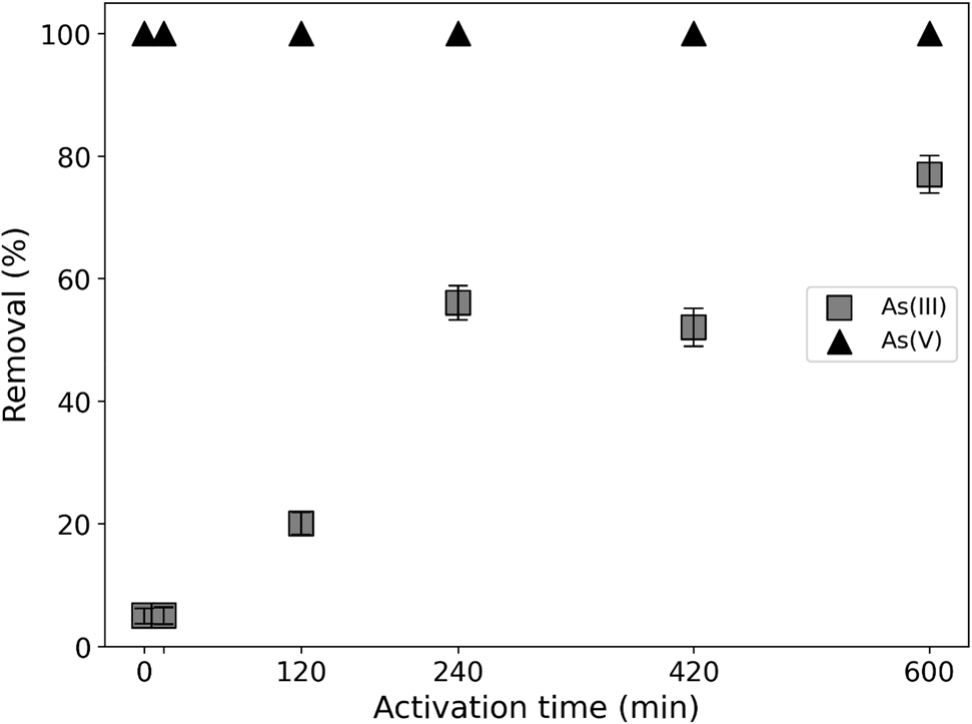
Removal efficiency of As(iii) and As(v) anions by adsorbents whit different activation times. Experimental conditions: pH 7, As(iii) and As(v) concentration: 1 mg L^−1^, adsorbent mass: 0.1 g, contact time: 60 min and 25 °C.

Fig. 9 shows the removal efficiency of arsenic for the non-activated adsorbent material and samples subjected to different times of mechanical activation. Firstly, a total elimination of the As(v) species can be observed regardless of the activation time. In the case of As(iii), it was observed that the removal efficiency is greater as the activation time increases, registering a removal percentage of the most activated material corresponding to 7.5 times that extracted by the non-activated material.

The results of this study, indicating that the adsorption of As(v) was greater than that of As(iii), are consistent with findings reported in the literature. For example, Lin and Puls (2000)^7^ evaluated the adsorption of arsenic species using various aluminosilicates (clay minerals) and observed that all six clays tested demonstrated higher adsorption capacity for As(v) compared to As(iii).

The increase in the performance of the activated samples in the removal of As(iii) in relation to the non-activated sample could be due to the smaller particle size. Previous studies confirm that the mechanical activation of different aluminosilicates, including feldspars, leads to a reduction in the size of the crystallites. As the particle size decreases, the surface area increases, which generates a greater number of available active sites that favor their interaction with the adsorbate.^40^ The unactivated sample (NA) and the sample with the longest activation time (A5) were selected to continue the evaluation of the effects of pH and adsorbent dose on As adsorption efficiency. In addition, it has been shown that the surroundings of the OH are easily modified with mechanical treatments, having a fundamental role in the adsorption of different analytes.^41^

#### 3.2.2. Effect of adsorbent concentration

**Fig. 10:**
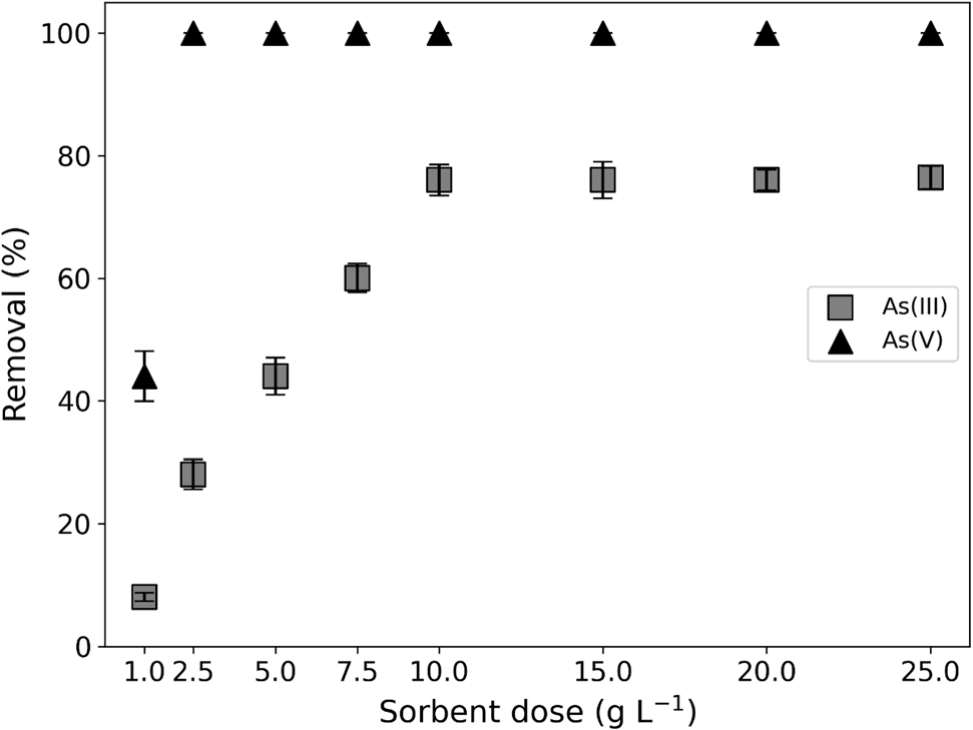
Effect of sorbent doses on As(iii) and As(v) removal percentage. Experimental conditions: As(iii)–As(v) initial concentration: 1 mg L^−1^, pH: 9, contact time: 60 min.

Removal efficiency for As(iii) and As(v) was studied *versus* the concentration of adsorbent for A5. Results were reported in Fig. 10. The effect of solid/liquid ratio in the batch reaction was studied to determine the optimum quantity of adsorbent required to remove arsenic. Increasing the doses of adsorbent enhanced the arsenic removal efficiency of both samples. In order to select the same adsorbent mass for As(iii) and As(v) and maximize adsorption efficiency an optimal working solid/liquid ratio of 10 g L^−1^ was selected.

Previous studies confirm that increasing the solid/liquid ratio will increase the number of surface sites available for sorption, resulting in higher ion removal efficiency.^42^ It is important to note that increasing the adsorbent amount from 10 g L^−1^ to 25 g L^−1^ did not enhance the removal efficiency. This may be due to the availability of more surfactant sites than ions in the solution.^43^ Furthermore, at a higher concentration of adsorbent, an interaction between the adsorbent particles may occur, favoring their aggregation. This would cause a decrease in the available active sites and, consequently, a decrease removal efficiency.^44^

#### 3.2.3. Effect of pH on removal efficiency

The adsorption of heavy metal ions is strongly influenced by the pH of a solution, making it a key factor in controlling the adsorption of ions onto the surface of aluminosilicates. Altering the pH can modify surface properties, thereby affecting ion removal efficiency.^43^

**Fig. 11:**
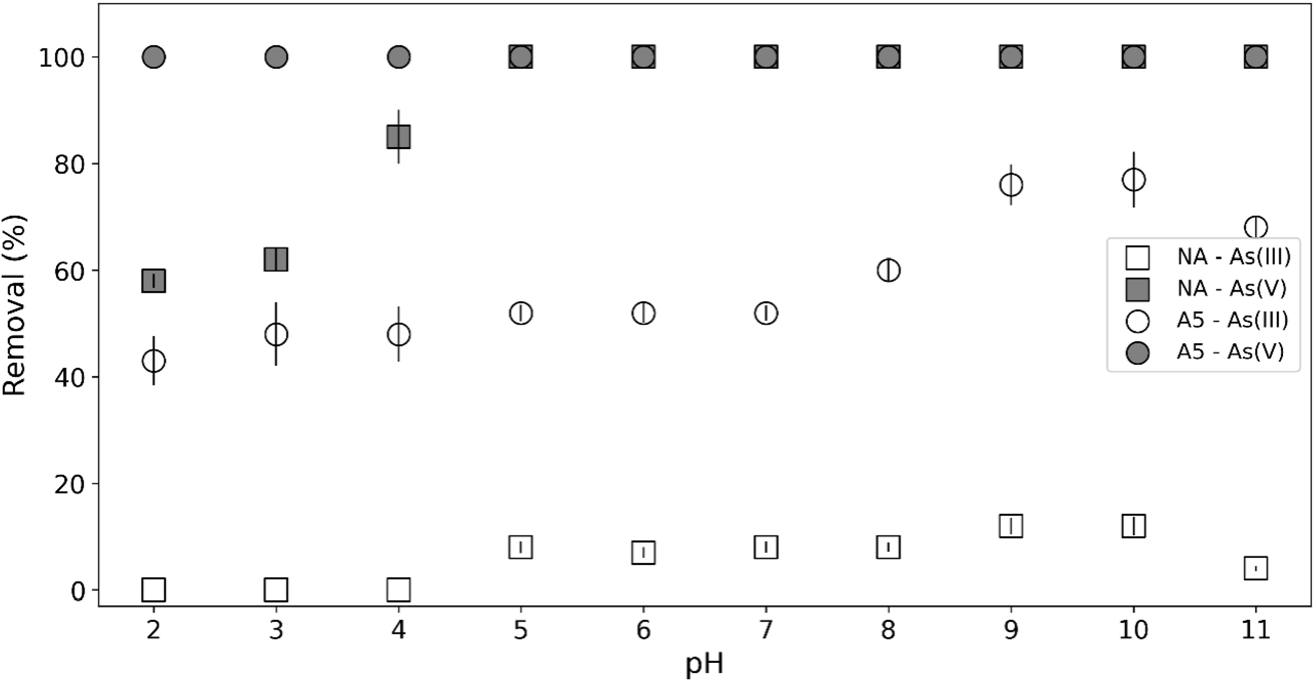
Effect of pH on As(iii) and As(v) adsorption onto the feldspar samples. Experimental conditions: As(iii) and As(v) initial concentration: 1 mg L^−1^, sorbent dose: 10 g L^−1^, contact time: 60 min.

Fig. 11 shows that the removal percentage of As(v) using the A5 material was 100%, regardless of the pH value, suggesting that the adsorption mechanism in this case is not strongly influenced by electrostatic interactions. This behavior can be correlated with Fig. 5, where the point of zero charge (pHpzc) of the materials is observed to be in the pH range of 5–7. In this interval, the adsorbent surface is neutral, minimizing possible electrostatic repulsion effects and favoring adsorption mechanisms such as ligand exchange.

For the remaining three cases, a general pattern is identified: at acidic pH values, the removal percentage of As(iii) is low and progressively increases as the pH becomes neutral or slightly basic. This behavior can be understood in terms of Fig. 6, which shows the electrophoretic mobility of the materials as a function of pH. The isoelectric point (IEP) is approximately 1.5, indicating that under strongly acidic conditions, the surface of the material has a significant positive charge. This favors the adsorption of anionic species such as As(v) but hinders the retention of As(iii), which in these conditions predominates in its neutral form (H_3_AsO_4_),^44^ reducing electrostatic interactions with the adsorbent.

From pH 11 onwards, a decrease in removal percentages is observed, which can be explained by the increasing negative charge of the adsorbent surface at higher pH values (Fig. 6), generating electrostatic repulsion with the anionic species of As(v) (HAsO_4_^2−^ and AsO_4_^3−^). Additionally, in this pH range, dissolution phenomena of the adsorbent material may occur, affecting its structural stability and reducing the availability of active sites for adsorption.

Taken together, the data suggest that the adsorption of As(v) on the A5 material is dominated by specific interactions rather than electrostatic effects, whereas the removal of As(iii) is strongly influenced by the surface charge of the adsorbent and its relationship with the speciation of arsenic in solution.

In the work of Tang and Dong (2022)^45^ it was shown that the release rates of Si and Al are significantly different in solutions with different pH values. This study, along with others, confirms that the release of Si and Al as a function of pH follows a U-shaped profile. The release of Si and Al is minimized in slightly acidic solutions (pH 5) and increases in either highly acidic (pH 1 and 2) or highly alkaline (pH 13) conditions. The stoichiometry of mineral surface dissolution can be examined by comparing the Al/Si ratio across solutions of varying pH levels. The Al/Si ratio of albite in an alkaline solution is similar to that corresponding to the atomic ratio in the crystal structure of albite, indicating that the solution is stoichiometric. For its part, the lowest Al/Si ratio in the solution was recorded at a slightly acid pH, coinciding with the value at which an increase in As extraction occurs in this work. At acidic pH this ratio is three times higher (non-stoichiometric ratio), which would indicate a greater release of Al into the liquid phase. The authors attribute the higher Al/Si ratios observed at acid pH to the formation of surface coatings, as well as to the higher dissolution capacity of Al in acid solutions.

#### 3.2.4. Adsorption mechanisms

**Fig. 12:**
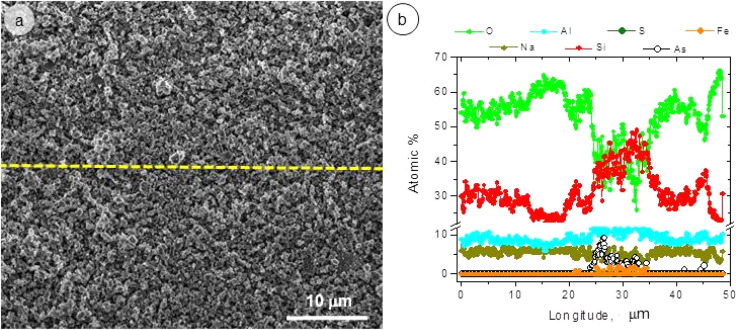
(a) SEM micrograph and (b) EDS line scan of A5 after As(v) adsorption.

The lower release of Al in the solution influenced the As adsorption process. The presence of Al in the adsorbent structure plays a fundamental role in As adsorption since it contributes to the presence of a greater number of surface Al–OH groups that favors ligand exchange reactions. This can be verified by observing Fig. 12, which corresponds to a SEM and EDS analysis of the sample after As(v) adsorption.

In Fig. 12, it can be seen that those areas where the Al content increases coincide with those where the As content is recorded. This allows us to infer that the Al plays a fundamental role in the adsorption of As.

Previous works in which feldspars were analyzed as adsorbents have shown that the higher As adsorption was correlated with the higher aluminum content.^44^ Similar reactions have been demonstrated in zeolites and phyllosilicates where terminal Al–OH groups develop at the edges that participate in As adsorption reactions.^42^

In the work of Yazdani *et al.* (2016)^44^ it is revealed that some feldspars show the ability to buffer highly acidic and basic solutions. In other words, starting from solutions with different pH values in the range of 3–11, solutions with pH values in the range of 4.5 to 7.5 were obtained. These results are in agreements with those found in this work (Fig. 5).

At the final pH values observed in this work (4–7) for As(iii) the predominant species is H_3_AsO_3_. Being a neutral species, it could be inferred that the determining adsorption mechanism is ligand exchange. This is demonstrated by other works where they propose that the main mechanism for the elimination of As(iii) would be chemisorption and that it adsorbs more favorably on non-ionized surface functional groups.^46^

For its part, As(v) is forming negative species, so at a final pH close to pHpzc the adsorption is greater since the surface is neutral instead of being negatively charged (decreasing adsorption due to electrostatic repulsions). In the case of the A5 material, the adsorption was 100% regardless of the pH value, so we can infer that in this case also ligand exchange is the dominant extraction mechanism.

Contrary to what was observed in our results, in Yazdani *et al.* (2016), As(v) adsorption is greater at acidic pH. This is because the surface is positively charged, while the As(v) species are negative, favoring coulombic interactions.^44^ On the other hand, On the other hand, at basic pH, repulsive forces occur because the surface is mainly negatively charged due to pH values higher than the pHpzc of the adsorbent, causing less adsorption.

Chemical bonds and electrostatic forces are involved in adsorption phenomena. The latter determine the forces of attraction or repulsion between the ions and the adsorbents. As has been observed in other works,^36^ it could be expected that at pH values below pHpzc the adsorption of As(v) is greater since the surface is positively charged and arsenic forms negatively charged species favoring coulombic interactions.

However, as discussed above, at acidic pH Al leaching can occur, leading to a decrease in adsorbate retention. The highest adsorption rates observed in this work occur in the pH range of 5–8. These values are close to the pHpzc values, that is, when the surface is neutral. Since the surface is uncharged, the repulsive forces are minimized favoring chemisorption.

Concluding, the literature suggests that in feldspars, the removal of As involves not only ligand exchange but also electrostatic attraction between negatively charged species and positively charged ≡Al–OH^+^ sites, along with chemical interactions.^42,44^ Nevertheless, we do not exclude the possible contribution of other mechanisms such as ion exchange.

#### 3.2.5. Adsorption kinetics

Kinetic studies in adsorption processes contribute to determine the speed in which adsorbates are extracted from the aqueous phase by the adsorbent. Being a time-dependent process, it is necessary to know its speed for the design, evaluation and comparison of adsorbents. Different kinetic models have been proposed to determine the adsorption rate and the equilibrium time for adsorption. Adsorption kinetic models are employed to determine how adsorption capacity varies over time and to comprehend the various mass transfer mechanisms involved in the adsorption process.^47^ These models that explain a complex phenomenon such as adsorption stand out for their simplicity in their application and their easy interpretation. Among the most used mathematical models are the Langmuir and Freundlich models, which were selected in this study not only because they are the most widely applied and straightforward, but also because they enable meaningful comparisons with results reported in the literature. Owing to their theoretical foundation and widespread use, these models provide a rigorous and standardized framework for assessing adsorption phenomena.

##### 3.2.5.1. Pseudo first order model (PSO)

Also known as the Lagergren model,^48^ it is described mathematically by the eqn (3).3
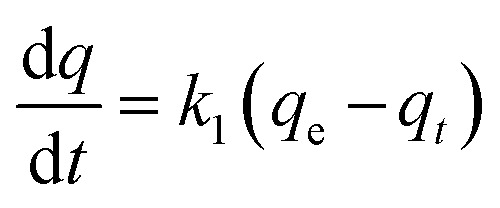
where *k*_1_ (min^−1^) is the first-order adsorption kinetic constant and *q*_e_ is the adsorbate charge at equilibrium. This parameter must be established before determining the fit of the model, considering the adsorbent initially free of solute, that is, under the boundary conditions *t* = 0 with *q*_*t*_ = 0 and *t* = *t* with *q*_*t*_ = *q*_*t*_. In an integrated form we have the eqn (4).

4 ln(*q*_e_ − *q*_*t*_) = ln*q*_e_ − *k*_1_*t*

This model considers that the driving force is the difference between the concentration of the adsorbed solute at equilibrium and the concentration of the adsorbed solute at a given time. It is based on the assumption that each adsorbate molecule/ion is assigned an adsorption site on the adsorbent material.

To determine the rate constants and metal absorption at equilibrium, linearization of the models is used by plotting log(*q*_e_ − *q*) *vs. t* of the equation. Subsequently, the acquired *q*_e_ value is contrasted against the experimental value^49^

##### 3.2.5.2. Pseudo second order model (PSO)

Also known as the Ho and McKay model,^50–52^ this model is usually attributed to the process that involves a chemisorption mechanism. It assumes that the rate at which active sites are occupied is proportional to the square of the number of vacant sites, as expressed in the eqn (5).5
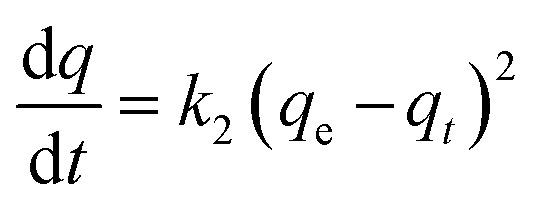
where *k*_2_ (g mg^−1^ min^−1^) is the second order adsorption kinetic constant, whose integration under the same boundary conditions, previously described, is represented by eqn (6).6
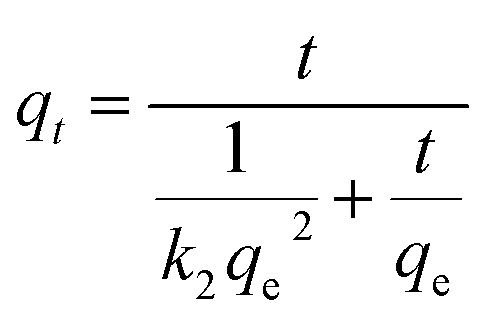


Pseudo second order rate constants are determined experimentally by plotting *t*/*q vs. t.*^49^

Adsorption kinetic experiments were conducted to examine the adsorption rate of As(iii) and As(v) at *T* = 25 °C. According to *R*^2^ values in Table 2, it could be seen that pseudo-second-order model fit better with experimental values compared to the other model, implying that the adsorption follows a pseudo-second-order rate mechanism.

**Table 2:** Parameters of kinetics models for the adsorption of As(iii) and As(v) onto A5

Adsorbate	Pseudo-first-order model			Pseudo-second-order model		
	*q*_e_ (mg g^−1^)	*k*_1_	*R* ^2^	*q*_e_ (mg g^−1^)	*k*_2_	*R* ^2^
As(iii)	0.0323	0.0013	0.893	0.0877	0.1119	0.999
As(v)	0.0018	0.0001	0.767	0.1983	0.3661	0.999

The modeling of kinetic data using the pseudo-second-order model indicated that the adsorption of arsenic in water onto the A5 adsorbent might involve a chemisorption process. These results were in agreement with other similar studies focus on As adsorption using different types of adsorbents.

Our results were in agreement other investigations where feldspars have been used. For example, in Yazdani *et al.* (2016)^44^ it was shown that the kinetic model that best fits the adsorption of As(v) on feldspars is that of PSO, since it presented better correlation values.

In Barraqué *et al.* (2021)^53^ the removal of As(v) in aqueous solution was studied using montmorillonite showing excellent agreement with PSO over the entire time interval. The adsorption kinetics of As(iii) and As(v) on a granular Mn-oxide-doped Al oxide adsorbent were evaluated, obtaining that the PSO model provided a better description of the adsorption process.^54^

Kinetic studies carried out by other authors also demonstrated that the removal of As(v) using aluminosilicates fits to a pseudo second order model.^55,56^

**Fig. 13:**
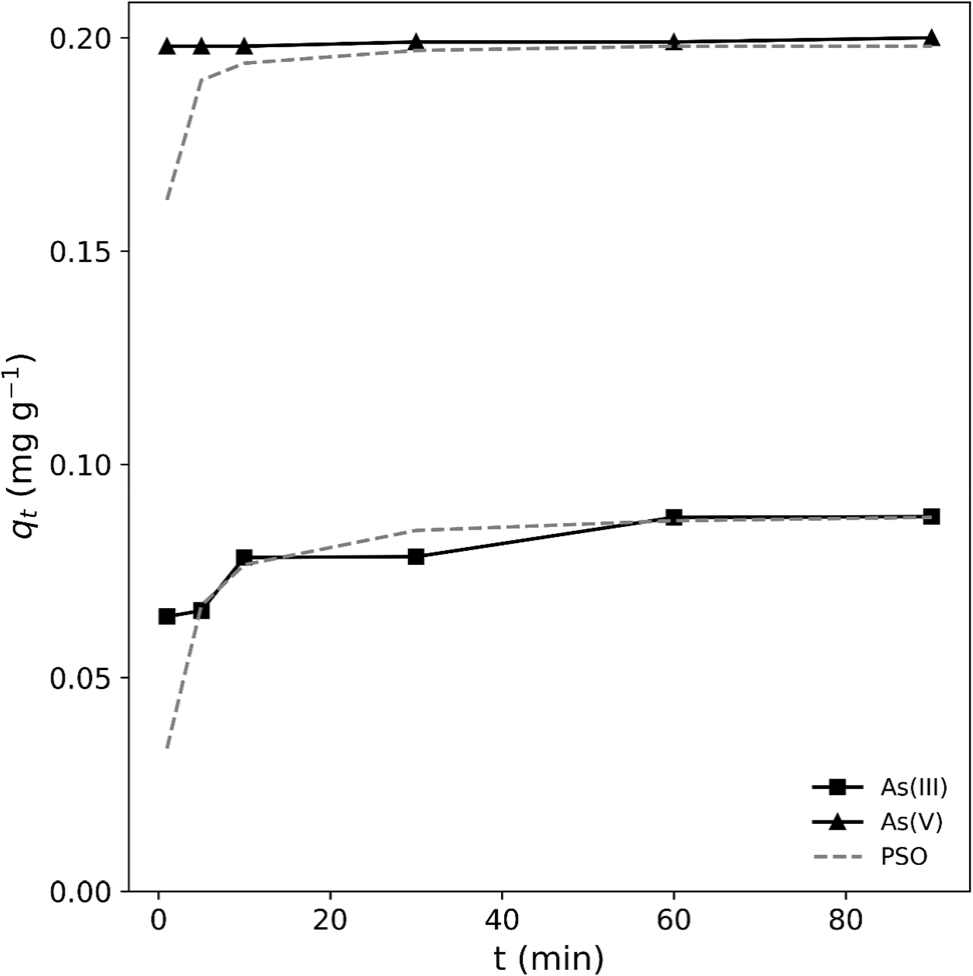
Experimental and theorical adsorption isotherm of As(iii) and As(v) onto A5.

Fig. 13 illustrates that adsorption of As(iii) and As(v) anions onto A5 occurs quite rapidly in the first 10 min, after which it slows down for both species. At the beginning of the adsorption process, the high adsorption rates suggest a large number of available surface sites, which gradually decreases as more sites become occupied by arsenic anions. Even though the pseudo-second-order model provided the best fit, this alone does not conclusively prove chemisorption. However, the co-localization of Al and As in EDS analyses (Fig. 12) and previous studies on ligand exchange with surface Al–OH groups strongly suggest a chemisorption mechanism.^25,43^

**Fig. 14:**
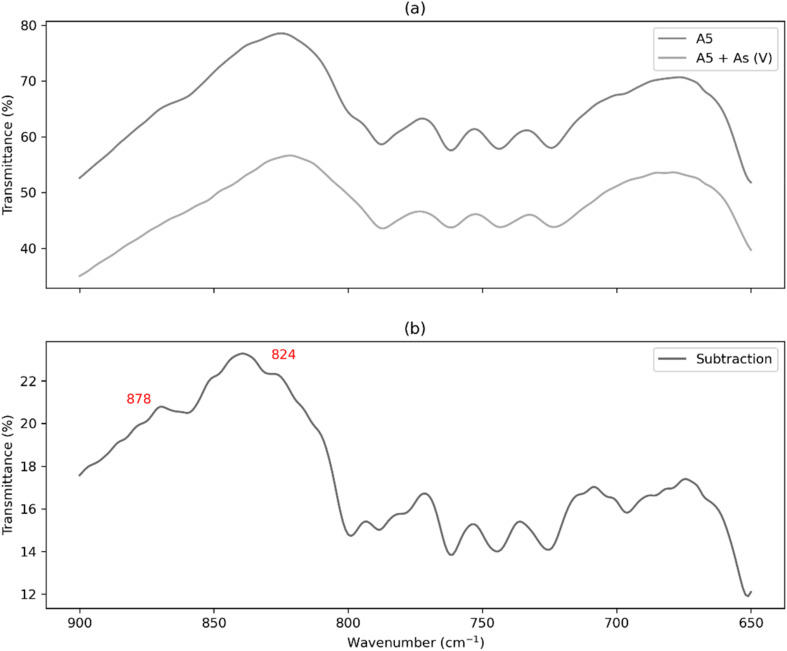
IR spectra (a) A5 sample (dark grey) and A5 sample after As sorption (light grey), (b) subtraction IR spectra of A5 sample and IR spectra of A5 sample after As sorption.

The observations described above are consistent with those shown in Fig. 14, which presents the FTIR spectra of the adsorbent material before and after adsorption (a), and the difference between both spectra (b). Due to the relatively low mass of As in the adsorbent, As–O bands are not distinctly visible. However, subtracting spectrum reveals the predominance of the 826 and 878 cm^−1^ bands, corresponding to AsO_4_^3−^(or protonated arsenate species, depending on the pH of the final solution), in agreement with previous findings in the scientific literature.^57^

##### 3.2.5.3. Adsorption isotherms

There are different models that graphically describe the amount of solute adsorbed by an adsorbent as a function of equilibrium concentration of the adsorbate.

##### 3.2.5.4. Langmuir isotherm

This two-parameter model, developed in 1916 by Irving Langmuir,^58,59^ proposes that the solid surface provides a fixed number of active sites for adsorption and the process takes place in a monolayer. Reached equilibrium, at any temperature and adsorbate concentration, a fraction of the active sites (*θ*) is occupied by the adsorbed species, and the other fraction (1 − *θ*) is free. It also considers that all the sites are equivalent to each other, so there is no interaction between the adsorbed species, and only one sorbate ion can bind in each active site.

The proposed mathematical model is:7
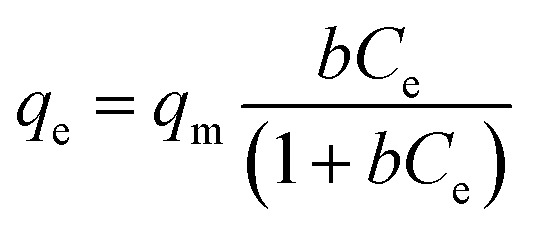
where *q*_e_ is the equilibrium sorption capacity, in mg g^−1^; *C*_e_ is the equilibrium concentration of the adsorbate in the solution, in mg L^−1^; *q*_m_ is the maximum sorption capacity for a complete monolayer, in mg g^−1^ and *b* is a constant related to the affinity between the particles of the adsorbent and the adsorbate, in L mg^−1^ whose value increases with the strength of the interaction.

##### 3.2.5.5. Freundlich isotherm

This model proposes that there is not a finite capacity of adsorption by the adsorbent. Furthermore, consider that the surface of the solid is heterogeneous and the active sites have different affinities. It assumes the adsorbate adsorption is carried out in multilayer.^60,61^

Mathematically it is expressed according to the following equation:8
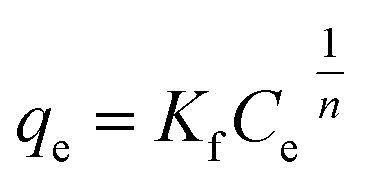
where *q*_e_ is the equilibrium sorption capacity, in mg g^−1^; *C*_e_ is the concentration of the metal at equilibrium, in mg L^−1^; *K*_f_ is the Freundlich constant related to the sorption capacity of the solid, in mg g^−1^; 1/*n* where *n* is a dimensionless constant that describes the affinity between adsorbent and adsorbate, therefore it is an indicator of the sorption intensity.

**Fig. 15:**
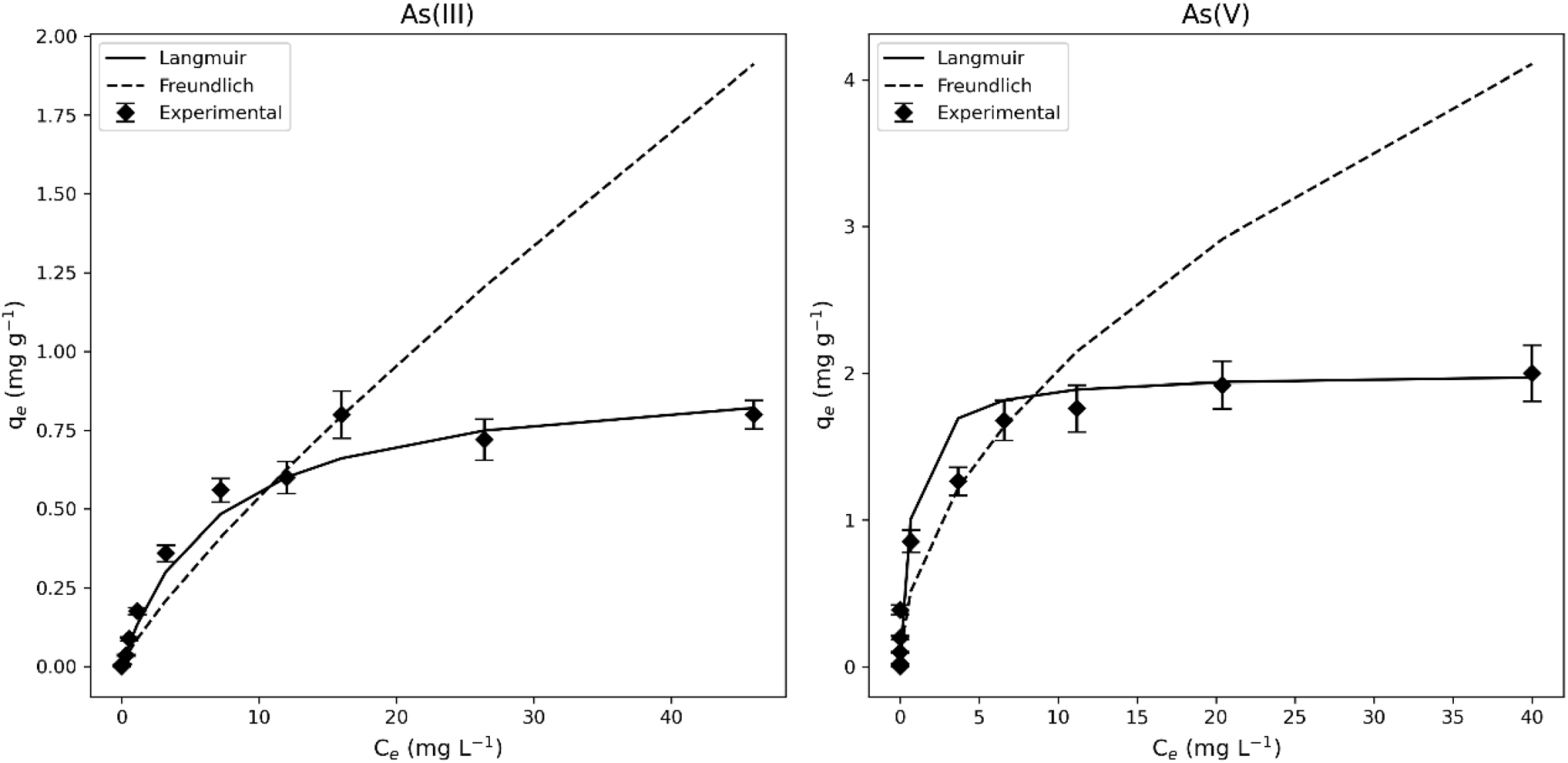
Isotherm of As(iii) and As(v) adsorption onto the A5 samples. Experimental conditions: contact time: 60 min, pH: 9, sorbent dose: 10 g L^−1^.

Table 3 shows the parameters obtained for the Langmuir and Freundlich isotherms, with the adsorption isotherms illustrated in Fig. 15. The results indicate that the Langmuir model provides a more accurate description of the adsorption isotherm compared to the Freundlich model, with *R*^2^ values of 0.953 and 0.997 for As(iii) and As(v), respectively. This suggests that the adsorption mechanism involves the formation of a monolayer of arsenic anions on the uniform sites of the adsorbent surface.^43^

**Table 3:** Parameters of isotherm models for the adsorption of As(iii) and As(v) onto A5

Adsorbate	Langmuir		Freundlich	
	*q*_m_ (mg g^−1^)	*R* ^2^	*n*	*R* ^2^
As(iii)	0.94	0.953	1.20	0.946
As(v)	2.00	0.997	1.96	0.854

The maximum adsorption capacity obtained from the data was found to be 0.8 mg g^−1^ for As(iii) and 2 mg g^−1^ for As(v) indicating that these materials are useful as potential adsorbent for the removal of As anions in water solution.

This behavior is in agreement with previously published works on As adsorption using aluminosilicates. In the research by Singh *et al.* (2002)^40^ adsorption capacity of feldspars for the treatment of water contaminated with As was evaluated. The results obtained were adjusted to the Langmuir model, suggesting the formation of a monolayer in the range of concentrations studied. In Chutia *et al.* (2009)^36^ As adsorption was carried out on synthetic zeolites in a range of concentrations from 10 to 150 mg L^−1^, obtaining high correlation values with the Langmuir model. In the study by Iriel *et al.* (2020)^56^ it was shown that the experimental data obtained from the adsorption of As(v) on iron-modified montmorillonite fit this same model.

Finally, we have included the adsorption capacity of different aluminosilicates used in arsenic removal in Table 4, where most of these substrates are permanently charged, unlike Albite/Nepheline (Fig. 6), which makes it more sensitive to pH variations. Although the adsorption capacity of synthetic albite/nepheline is modest compared with highly porous materials such as montmorillonite or zeolites, its normalized performance (*Q*_max_/*S*_BET_) is remarkable. This efficiency is attributed to the high density of surface Al–OH groups. In addition, the valorization of lithium extraction by-products represents a unique advantage over conventional aluminosilicates, aligning with circular economy principles.

**Table 4:** Comparison of As adsorption efficiency by various adsorbents

Aluminosilicate	Adsorbate	*q*_m_ (mg g^−1^)	*S*_BET_ (m^2^ g^−1^)	*Q*_max_/*S*_BET_	Reference
Feldspars	As(iii)	0.30	10.25	0.0293	40
Feldspars	As(v)	0.50	0.70	0.7692	25
Clay minerals	As(v)	0.07	156.60	0.0004	8
Montmorillonite	As(v)	9.00	63.00	0.1429	51
Montmorillonite	As(v)	6.30	107.00	0.0589	54
Clay minerals	As(iii)	7.00	73.10	0.0958	60
Albite/nepheline	As(iii)	0.94	8.30	0.1133	This work
	As(v)	2.00	8.30	0.2410	

#### 3.2.6. Application in water treatment

According to the scientific literature, the application of natural feldspars has been studied in different fields such as glass and ceramics industry. However, the study of the potential of synthetic feldspars and their possible applications has not been deepened.^23^

Arsenic concentrations in freshwater vary depending on the source, mobility, and the local geographic environment.^36^ In Argentina, although concentrations of up to 14 000 μg L^−1^ have been recorded, most studies indicate arsenic concentrations in the range of 0.05–2000 μg L^−1^.^13^ Due to this, it is essential to evaluate the application of adsorbents in this range of concentrations. In turn, the pH is a determining factor of the surface characteristics of the adsorbent and of the speciation of the adsorbates. Considering that the standard range of pH in drinking water varies from 6.5 to 8.5, it is necessary to investigate the removal of arsenic in these values.^36^

In Chutia *et al.* (2009),^36^ the arsenic adsorption capacity was evaluated using zeolites. Working with 2 mg L^−1^ solutions, it was shown that the adsorbents reduce the arsenic concentration below the reference value established by the WHO of 0.05 mg L^−1^ of As for drinking water. Similar results were obtained in Barraqué *et al.* (2021)^53^ where the studied materials presented removal percentages close to 100 and 90% for initial concentrations of 0.1 mg L^−1^ and 0.5 mg L^−1^ respectively. According to the authors, these results allow the application of these adsorbents in the removal of As due to the typical concentration range of arsenic in natural waters of Argentina and Latin America. The results derived from our work confirm the applicability of the adsorbents studied in the removal of arsenic in aqueous solution. Starting from initial concentrations in the range of 0.01 mg L^−1^ to 5 mg L^−1^, the A5 adsorbent allows the extraction of As(v) at rates higher than 90%, decreasing and close to 50% for As(iii). In particular, the application of these materials in the concentration range of 0.01–0.50 mg L^−1^ allows reducing As(v) levels to concentrations below 0.01 mg L^−1^, a value established by the WHO.

## 4. Conclusions

From the results obtained in this work we can highlight:

• Synthetic feldspars are suitable materials for use as adsorbents in the removal of arsenic in aqueous matrix.

• The application of these materials with a circular economy approach is highlighted, since they are obtained as by-products of lithium extraction from spodumene.

• The pH values in which the studied materials present better extraction values coincide with the typical pH range of drinking water.

• The materials show high percentages of As removal, especially for As(v) within the range of typical As concentrations recorded in water.

• Despite their low BET surface area, synthetic albite and nepheline exhibit a high density of Al–OH sites, enabling >90% arsenic removal under typical conditions. This demonstrates their efficiency as adsorbents and highlights a sustainable valorization pathway.

• The As adsorption capacity of the materials studied in the concentration ranges analyzed allows their application not only in the remediation of contaminated water but also with analytical applications.

## Conflicts of interest

The authors declare that they have no competing interests.

## Data Availability

The data supporting the findings of this study have been deposited in Zenodo and are openly available at https://doi.org/10.5281/zenodo.17144560.
